# A Descriptive Analysis of Direct Oral Anticoagulant Drugs Dosing Errors Based on Spontaneous Reports from the EudraVigilance Database

**DOI:** 10.3390/ph16030455

**Published:** 2023-03-17

**Authors:** Claudiu Morgovan, Carmen Maximiliana Dobrea, Adriana Aurelia Chis, Anca Maria Juncan, Anca Maria Arseniu, Luca Liviu Rus, Felicia Gabriela Gligor, Simona Alexandrina Ardelean, Laurentiu Stoicescu, Steliana Ghibu, Adina Frum

**Affiliations:** 1 Preclinical Department, Faculty of Medicine, “Lucian Blaga” University of Sibiu, 550169 Sibiu, Romania; claudiu.morgovan@ulbsibiu.ro (C.M.); carmen.dobrea@ulbsibiu.ro (C.M.D.); adriana.chis@ulbsibiu.ro (A.A.C.); ancamaria.juncan@ulbsibiu.ro (A.M.J.); felicia.gligor@ulbsibiu.ro (F.G.G.); adina.frum@ulbsibiu.ro (A.F.); 2 Department of Pharmaceutical Sciences, Faculty of Pharmacy, “Vasile Goldis” Western University of Arad, 310025 Arad, Romania; simonaaardelean@yahoo.com; 3 Department of Cardiology, Vth Medical Clinic, Faculty of Medicine, “Iuliu Haţieganu” University of Medicine and Pharmacy, 400139 Cluj-Napoca, Romania; laurentiu.stoicescu@umfcluj.ro; 4 Department of Pharmacology, Physiology and Pathophysiology, Faculty of Pharmacy, “Iuliu Haţieganu” University of Medicine and Pharmacy, 400349 Cluj-Napoca, Romania; stelianaghibu@yahoo.com

**Keywords:** DOACs, EudraVigilance, dosage errors, underdose, overdose, safety profile

## Abstract

Direct oral anticoagulant drugs (DOACs) interfere with the coagulation process, thus improving patient care for those who require anticoagulant treatment. This study presents a descriptive analysis of adverse reactions (ADRs) attributed to DOAC dosage errors (overdose, underdose, and improper dose). The analysis was performed based on the Individual Case Safety Reports from the EudraVigilance (EV) database. Results show that data reported for rivaroxaban, apixaban, edoxaban, and dabigatran are mostly regarding underdosing (51.56%) compared to overdosing (18.54%). The most dosage error reports were identified for rivaroxaban (54.02%), followed by apixaban (33.61%). Dabigatran and edoxaban had similar percentages (6.26% and 6.11%, respectively) regarding dosage error reports. Since coagulation issues can become life-threatening events, and factors such as advanced age and renal failure can influence the pharmacokinetics of drugs, the correct usage of DOACs is of utmost importance for the management and prevention of venous thromboembolism. Thus, the collaboration and the complementarity of knowledge of physicians and pharmacists may offer a reliable solution for DOAC dose management and improve patient care.

## 1. Introduction

Conversion of fibrinogen into fibrin driven by enzymes (active factors) and leading to the formation of thrombi is named coagulation. It involves intrinsic, extrinsic and common pathways [1,2]. Virchow’s triad (endothelial injury, hypercoagulability, and blood stasis) is responsible for the coagulation activity [3,4]. The risk factors of coagulation are represented by different physiological conditions (advanced age, pregnancy, prolonged immobilization, long vehicle rides or flights, etc.), pathologies (atrial fibrillation—AF, diabetes, obesity, surgery, cancer, varicose veins, congestive heart failure, renal failure, etc.) or drug use (contraceptive drugs, glucocorticoids, antidepressants, etc.) [5,6,7,8,9,10,11,12,13].

Anticoagulant drugs (AC) are necessary for preventing or treating venous thromboembolism. After the introduction of heparin in therapy, other ACs have also been used: vitamin K antagonists (VKAs), low molecular weight heparins (LMWHs), fondaparinux, etc. Subsequently, a new class of ACs (direct oral anticoagulant drugs—DOACs) was launched on the market in 2008. Currently, DOACs are represented by two categories: direct oral thrombin inhibitors (dabigatran) and direct oral factor Xa inhibitors (rivaroxaban, apixaban, edoxaban, and betrixaban) [14,15,16,17,18,19,20]. The main indications of DOACs are: stroke prevention in nonvalvular AF and the prevention or treatment of venous thromboembolism, including after total knee replacement and after total hip replacement [21]. Compared to other ACs, some advantages are conferred by DOACs: increased adherence due to oral administration, rapid effect and fixed doses, limited interindividual variation, decreased risks of interactions or adverse effects (e.g., bleeding), etc. [22,23,24]. Therefore, DOACs have better safety and efficacy profiles, even in elderly patients [25]. The existence of specific reversal agents (adexanet for rivaroxaban and apixaban; idarucizumab for dabigatran) and promising preliminary results of a general reversal agent (ciraparantag) are also encouraging [26,27]. Their effectiveness and their toxicity, especially the higher bleeding risk, depend on the correct dosage. Thus, the use of appropriate doses for each patient is important. Therefore, patient follow-up and dosage adjustment according to age, body weight, drug–drug interactions or medical condition (renal/hepatic impairment, risk of bleeding, etc.) for each patient must be performed [28]. To reduce their risk of bleeding, underdosing has been reported as a common practice, especially in elderly patients. Hence, the protection against thromboembolic events and their efficacy are reduced [25,29,30,31]. In addition, underdosing has been associated with increased cardiovascular hospitalization [32]. Different studies have reported off-label underdosing of DOAC used for reducing the risk of stroke. However, underdosing presented increased all-cause mortality, and did not reduce the risk of bleeding or embolization [30,31,33]. In addition, a fivefold increased risk of stroke in patients with AF and renal failure who received underdosed apixaban was observed [28]. On the other hand, DOAC overdose increased all-cause mortality and the severity of hemorrhagic events. Overdosing is less common than underdosing in most studies [32,34,35]. Moreover, no statistically significant efficacy was observed in patients with renal disease [36].

According to the European Medicines Agency (EMA), medication errors (ME) represent unintended mistakes that occur during drug treatment. Regardless of the cause, whether resulting from inappropriate prescribing, storage, dispensing, preparation, or administration, ME can lead to adverse reactions (ADRs). As MEs represent a major public health problem, national pharmacovigilance systems from the European Union must collect and report them. In this context, the EMA elaborated a “good practice guide on recording, coding, reporting and assessment of medication errors” and a “good practice guide on risk minimization and prevention of medication errors”. These instruments support the activity of national competent authorities and all stakeholders involved in the pharmaceutical sector. According to these regulations, MEs are human- or process-mediated failures and do not refer to intentional overdose, off-label use, misuse, and abuse of the drug. All collected MEs must be reported and centralized in the EudraVigilance (EV) database, regardless of whether the error is associated with ADRs [37,38,39]. EV is the public system for collecting suspected ADRs to drugs that have been authorized or included in clinical trials in the European Economic Area (EEA), and it is managed by the EMA. The database has been in use since 2001 and includes Individual Case Safety Reports (ICSR) voluntarily submitted by health professionals, patients, and other public categories [40,41,42]. EV combines multiple functions such as data collection, management and analysis and employs standard terminology. All operations are subjected to security management. This reliable database facilitates the rapid identification of possible safety concerns. The system detects and removes duplicates. The resulting European database of suspected adverse drug reaction reports allows for open access to up-to-date information [42]. On the other hand, marketing authorization holders and sponsors of clinical trials have the obligation to transmit suspected ADRs to national competent authorities [40,41,42].

Because of their increased use in clinical practice, DOACs must have a favorable benefit/risk profile. The spontaneous reporting system, EV, could quickly offer a characterization of this profile in order to improve the quality of patients’ life. Thus, the aim of the present study is the evaluation of the reporting frequency of dosing errors associated with DOACs. We performed a retrospective analysis of ADRs related to these errors reported to the EV database. A descriptive analysis and a disproportionality study of these spontaneous reports were undertaken. The effectiveness and the toxicity of DOAC, especially the higher bleeding risk, depends on the correct dosage. Thus, the use of appropriate doses for each patient is important.

## 2. Results

### 2.1. Descriptive Analysis

A total number of 427,318 reported ADRs in EV were identified for 24 AC drugs (30.7% rivaroxaban, 16.48% apixaban, 13.49% dabigatran, and 2.18% edoxaban). The total number of ADRs reported for DOACs was 268,551 (62.85% of total ADRs reported for AC) (Table 1).

**Figure 1:**
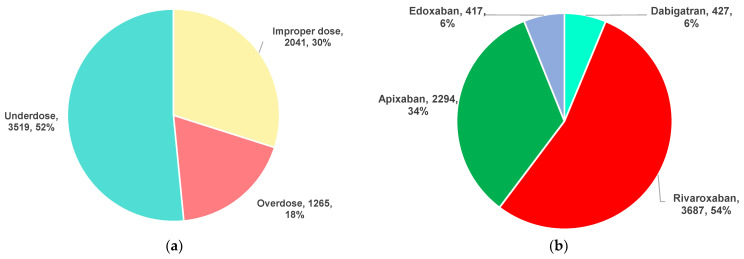
Distribution of DOAC dosing errors reported in EV: (**a**) by category; (**b**) by drug.

**Figure 2:**
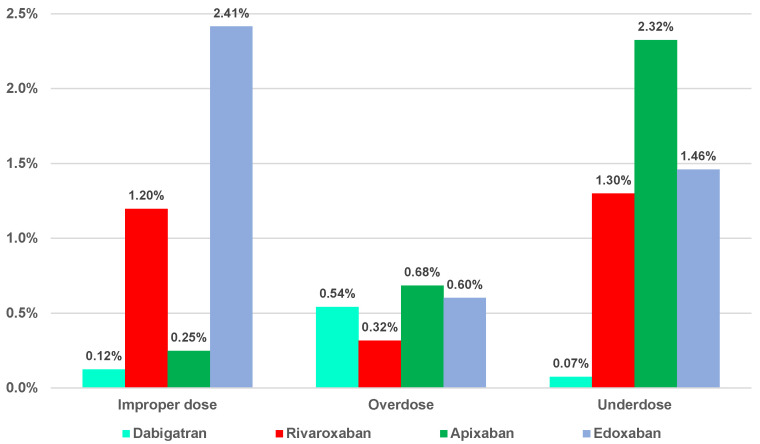
Distribution of the three categories of dosing errors from the total reports.

The distribution of DOAC dosing errors by category (overdose, underdose and improper dose, the latter including those PTs referring to dosing error, but not associated with overdosing or underdosing) and drug is represented in Figure 1. Most of the ADRs were related to DOAC underdosing (52%). Rivaroxaban (54%) and apixaban (34%) were associated with the highest number of ICSR reported in EV (Figure 2).

The performed analysis showed the highest percentages of reported improper dose errors from the total ADRs for edoxaban (2.41%) and rivaroxaban (1.20%). The proportion of overdosing errors from the total ADRs was approximatively equal for apixaban (0.68%), rivaroxaban (0.60%), and dabigatran (0.54%), and was slightly lower for rivaroxaban (0.32%). Regarding the underdosing errors of total ADRs, the highest value was noted for apixaban (2.32%), and a small percentage was observed for dabigatran (0.07%) (Figure 2).

**Figure 3:**
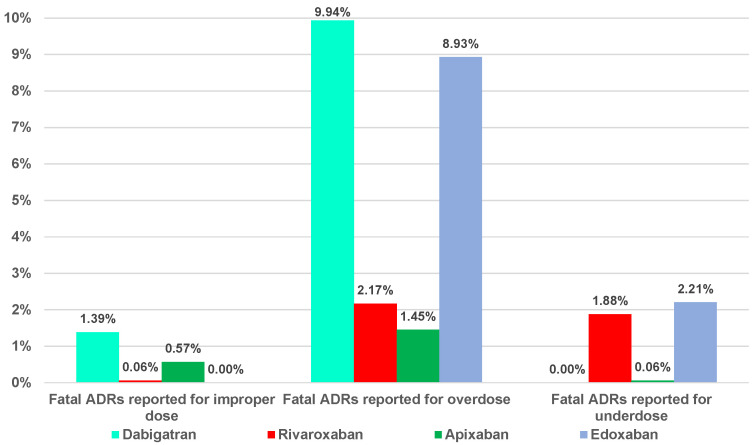
Distribution of the fatal ADRs related to DOACs dosing errors.

Of the total dosing errors, a small part was represented by ADRs that had a fatal outcome. For ADRs related to improper dose errors, the range was between 0% (edoxaban) and 1.39% (dabigatran), and for underdosing errors, it was between 0% (dabigatran) and 2.21% (edoxaban). Overdosing errors presented high percentages of the total reported ADRs related to each DOAC (1.45%—apixaban, 2.17%—rivaroxaban, 8.93%—edoxaban, and 9.94%—dabigatran) (Figure 3).

**Figure 4:**
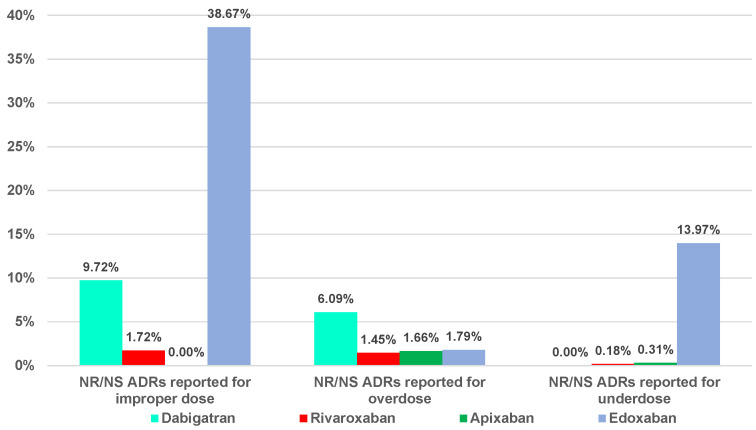
Distribution of the “not recovered (NR)/not resolved (NS)” ADRs related to DOACs dosing errors.

A high proportion of not recovered (NR)/not resolved (NS) cases related to dosing errors was reported for edoxaban (38.67%—improper dose errors, 13.97%—underdosing errors) and dabigatran (9.72%—improper dose errors and 6.09%—overdosing errors) (Figure 4).

No significant correlation was observed between each analyzed ADR category and the total ADRs reported in EV for DOACs (Table 2).

### 2.2. Disproportionality Analysis

#### 2.2.1. Overdosing Errors

**Figure 5:**
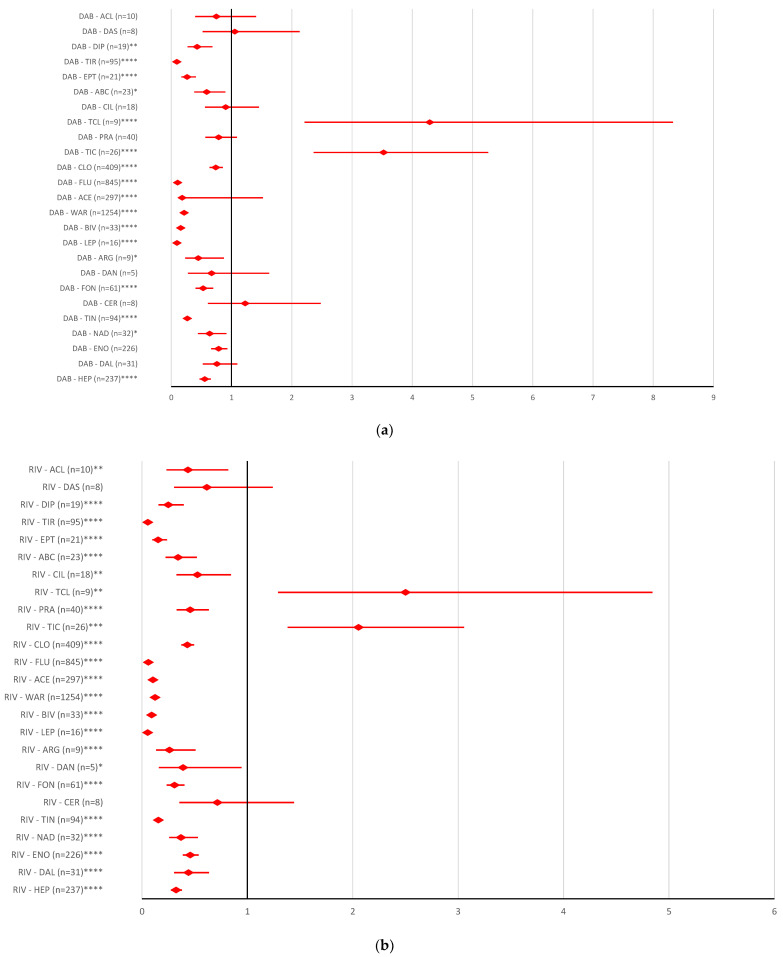

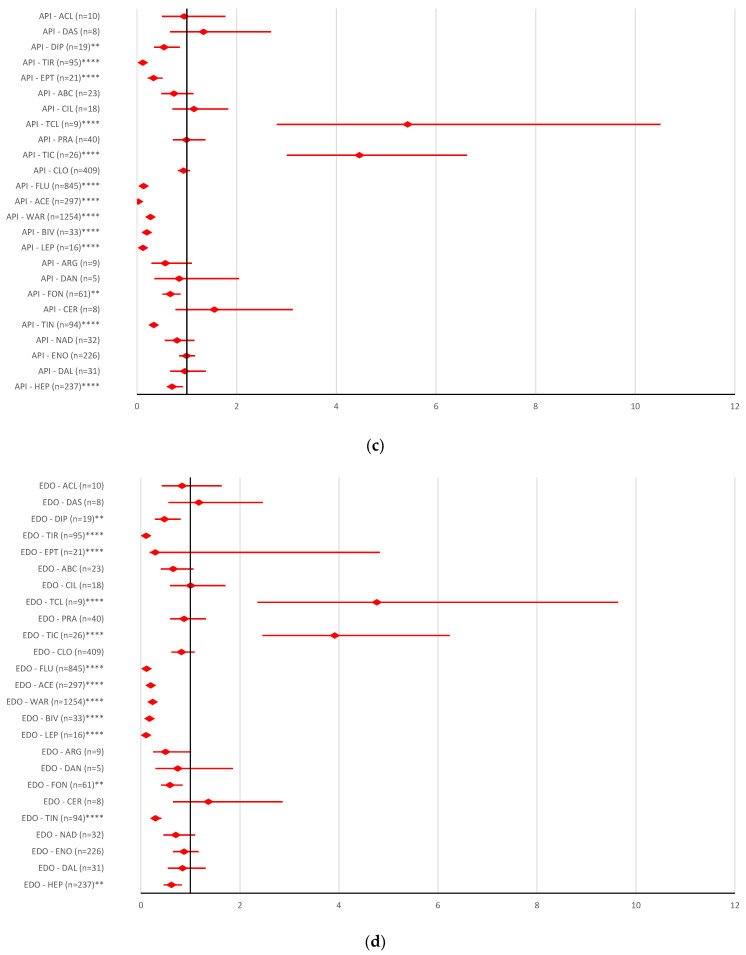
Reporting odds ratio of ADRs referring to DOAC overdose: (**a**) dabigatran—AC and antiplatelet drugs; (**b**) rivaroxaban—AC and antiplatelet drugs; (**c**) apixaban—AC and antiplatelet drugs; (**d**) edoxaban—AC and antiplatelet drugs. ABC—abciximab; ACL—combination of acetylsalicylic acid and clopidogrel; ACE—acenocoumarol; API—apixaban; ARG—argatroban; BEM—bemiparin; BIV—bivalirudin; CER—certoparin; CIL—cilostazol; CLO—clopidogrel; DAB—dabigatran; DAL—dalteparin; DAN—danaparoid; DAS—combination of dipyridamole and acetylsalicylic acid; DIP—dipyridamole; EDO—edoxaban; ENO—enoxaparin; EPT—eptifibatide; FLU—fluindione; FON—fondaparinux; HEP—unfractionated heparin; LEP—lepirudin; NAD—nadroparin; PRA—prasugrel; RIV—rivaroxaban; TCL—ticagrelor; TIC—ticlopidine; TIN—tinzaparin; TIR—tirofiban; WAR—warfarin; * *p* <0.05; ** *p* ≤ 0.01; *p* ≤ 0.001; **** *p* ≤ 0.0001.

All DOACs had a high reporting probability of ADRs involving overdose only compared to ticagrelor (dabigatran: ROR 3.5245, 95% CI 2.3615–5.2603; rivaroxaban: ROR 2.0548, 95% CI 1.3821–3.0549; apixaban: ROR 4.4619, 95% CI 3.006–6.6231; edoxaban ROR 3.9147, 95% CI 2.4569–6.2374) and ticlopidine (dabigatran: ROR 4.2896, 95% CI 2.2101–8.3256; rivaroxaban: ROR 2.5008, 95% CI 1.2915–4.8424; apixaban: ROR 5.4304, 95% CI 2.8071–10.5053; edoxaban ROR 4.7644, 95% CI 2.3552–9.6382) (Figure 5).

#### 2.2.2. Underdosing Errors

**Figure 6:**
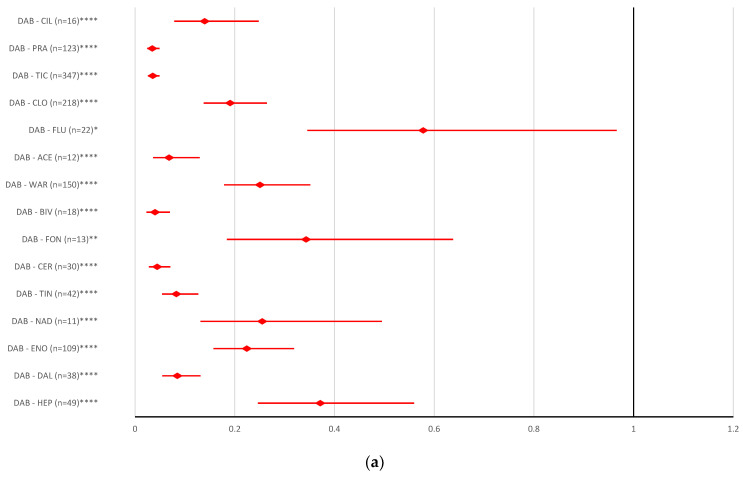

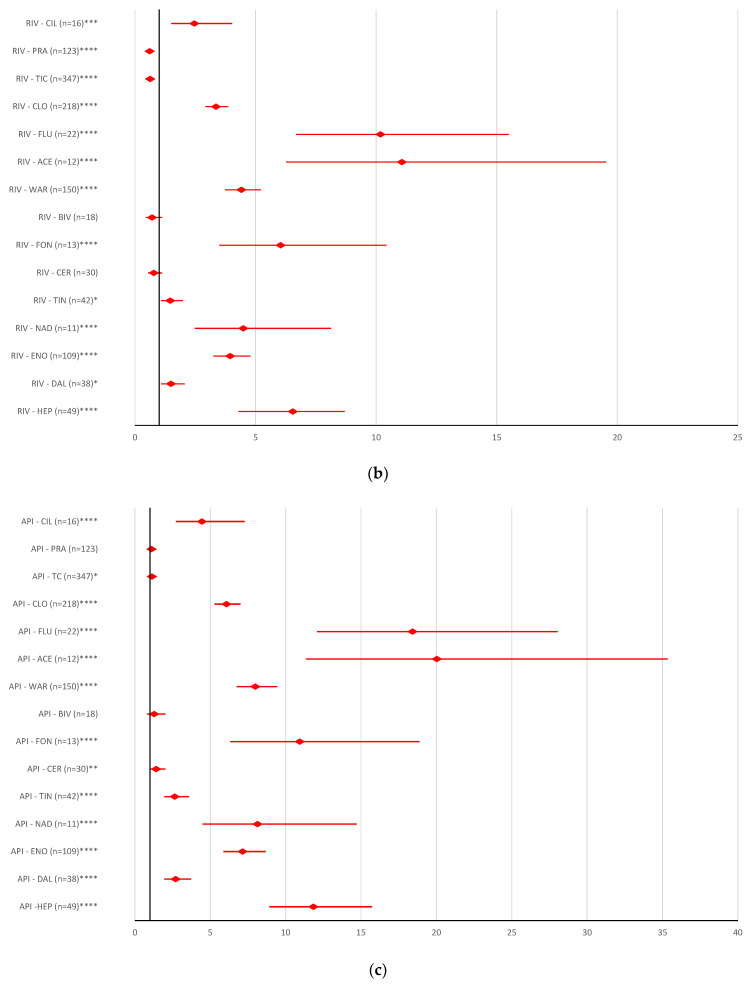

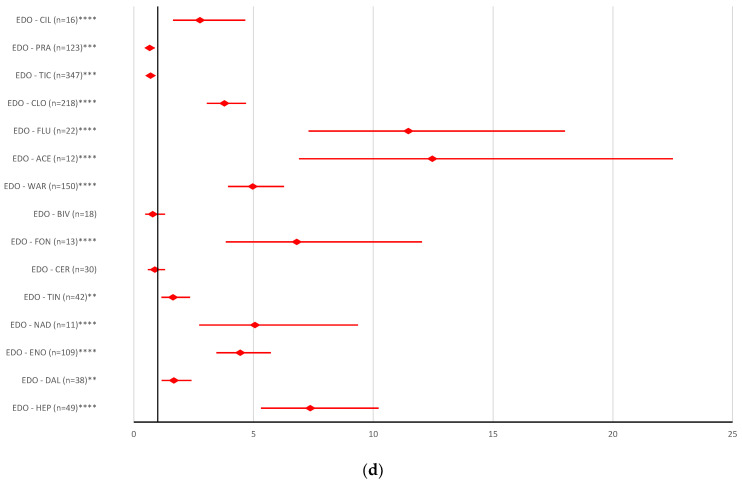
Reporting odds ratio of ADRs referring to DOAC underdose: (**a**) dabigatran—AC and antiplatelet drugs; (**b**) rivaroxaban—AC and antiplatelet drugs; (**c**) apixaban—AC and antiplatelet drugs; (**d**) edoxaban—AC and antiplatelet drugs. ABC—abciximab; ACL—combination of acetylsalicylic acid and clopidogrel; ACE—acenocoumarol; API—apixaban; ARG—argatroban; BEM—bemiparin; BIV—bivalirudin; CER—certoparin; CIL—cilostazol; CLO—clopidogrel; DAB—dabigatran; DAL—dalteparin; DAN—danaparoid; DAS—combination of dipyridamole and acetylsalicylic acid; DIP—dipyridamole; EDO—edoxaban; ENO—enoxaparin; EPT—eptifibatide; FLU—fluindione; FON—fondaparinux; HEP—unfractionated heparin; LEP—lepirudin; NAD—nadroparin; PRA—prasugrel; RIV—rivaroxaban; TCL—ticagrelor; TIC—ticlopidine; TIN—tinzaparin; TIR—tirofiban; WAR—warfarin; * *p* <0.05; ** *p* ≤ 0.01; *p* ≤ 0.001; **** *p* ≤ 0.0001.

Regarding the underdosing ADRs, it was noted that the reporting probability for dabigatran was not significantly different from other ACs or antiplatelet drugs (Figure 6a). All other DOACs presented a high probability of reporting ADRs related to underdosing errors, except for certoparin (rivaroxaban: ROR 0.7808, 95% CI 0.5426–1.1237; apixaban: ROR 1.4128, 95% CI 0.9816–2.0335; edoxaban: ROR 0.8793, 95% CI 0.5903–1.3099); bivalirudin (rivaroxaban: ROR 0.7044, 95% CI 0.4408–1.1256; apixaban: ROR 1.2745, 95% CI 0.7975–2.0369; edoxaban: ROR 0.7931, 95% CI 0.483–1.3027), ticlopidine (rivaroxaban: ROR 0.6259, 95% CI 0.557–0.7033, edoxaban: ROR 0.7049, 95% CI 0.5771–0.8608), and prasugrel (rivaroxaban: ROR 0.608, 95% CI 0.5054–0.7315; apixaban: ROR 1.1001, 95% CI 0.9141–1.325; edoxaban: ROR 0.6647, 95% CI 0.5353–0.8758) (Figure 6b–d).

#### 2.2.3. Improper Dose Errors

**Figure 7:**
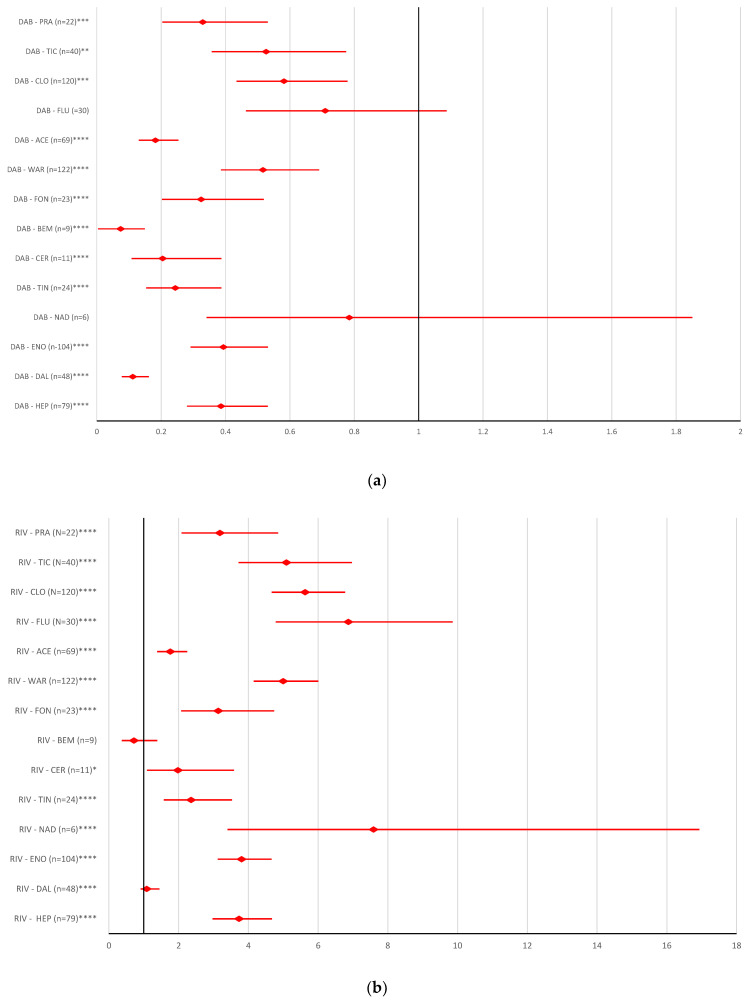

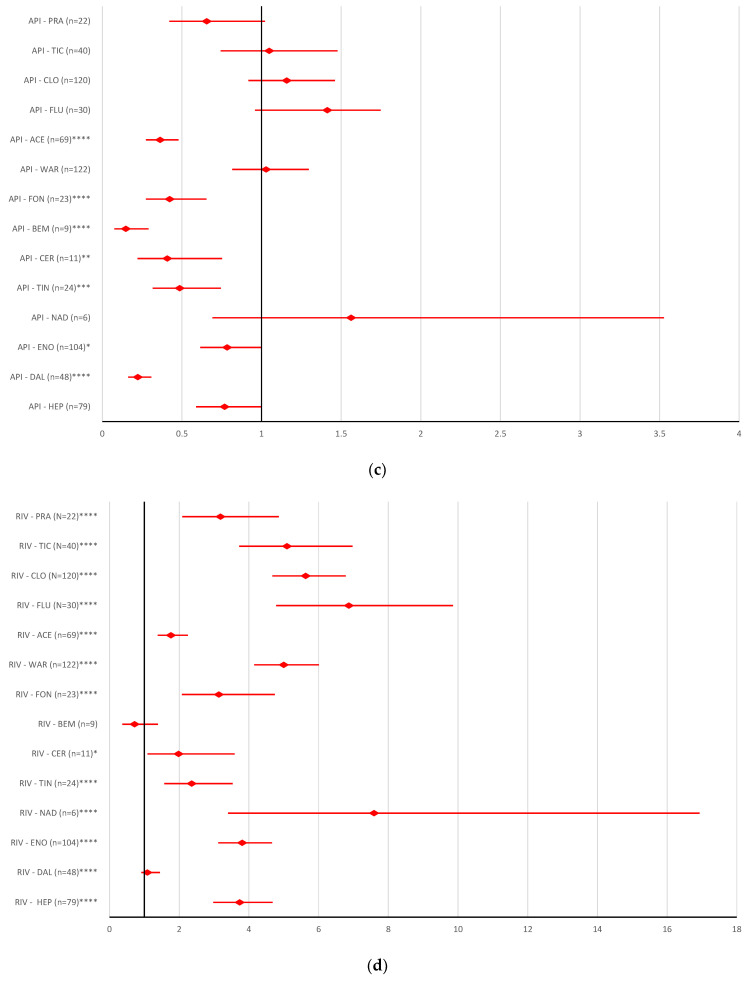
Reporting odds ratio of ADRs referring to DOAC improper dose errors: (**a**) dabigatran—AC and antiplatelet drugs; (**b**) rivaroxaban—AC and antiplatelet drugs; (**c**) apixaban—AC and antiplatelet drugs; (**d**) edoxaban—AC and antiplatelet drugs. ACE—acenocoumarol; API—apixaban; BEM—bemiparin; CER—certoparin; CLO—clopidogrel; DAB—dabigatran; DAL—dalteparin; EDO—edoxaban; ENO—enoxaparin; FLU—fluindione; FON—fondaparinux; HEP—unfractionated heparin; NAD—nadroparin; PRA—prasugrel; RIV—rivaroxaban; TIC—ticlopidine; TIN—tinzaparin; WAR—warfarin; * *p* <0.05; ** *p* ≤ 0.01; *p* ≤ 0.001; **** *p* ≤ 0.0001.

In comparison with AC drugs from other classes and antiplatelet drugs, dabigatran and apixaban had a low reporting probability for ADRs referring to improper dosing errors (Figure 7a,c). On the other hand, for rivaroxaban, a high reporting probability for improper dosing errors was observed, except for dalteparin (ROR 1.0839, 95% CI 0.912–1.4469) and bemiparin (ROR 0.7168, 95% CI 0.3703–1.388) (Figure 7b). In addition, edoxaban had a higher reporting probability for this category of ADRs, except for bemiparin (ROR 1.4654, 95% CI 0.7484–2.8694) (Figure 7d).

## 3. Discussion

This study presents a descriptive analysis of MEs reported in the EV database related to DOAC doses. From a total of 91 preferred terms (PTs) related to dosing error, reports in EV were identified for 24 PTs. These PTs were classified in overdosing, underdosing or improper dose errors. Until 3 December 2022, the total number of reports registered in EV for four DOACs (dabigatran, rivaroxaban, apixaban, and edoxaban) was 268,551. Betrixaban is not registered in the EU by EMA, and no ICSR was found for betrixaban in EV [43]. No ICSR was found for betrixaban; 48.85% of them were associated with rivaroxaban (*n* = 131,182), 26.22% with apixaban (*n* = 70,424), 21.46% with dabigatran (*n* = 57,627), and 3.47% with edoxaban (*n* = 9318).

A study performed by Camm et al. included 10,426 patients that received DOAC treatment (dabigatran, apixaban, rivaroxaban, edoxaban). Of the total patients, 23.2% were underdosed, and 3.8% were overdosed. The most frequent dosing ADRs were reported for edoxaban (40.6%). For the other three drugs, the proportions varied between 70% and 82% (81.9% for dabigatran, 72.4% for rivaroxaban, and 70.1% for apixaban). The proportion of underdosed patients was very high in the total of patients treated with edoxaban (55.9%) and was high in patients treated with apixaban (28.7%), rivaroxaban (21.1%) and dabigatran (15.8%). Regarding the overdoses, the same study showed smaller proportions of these ADRs compared to underdoses (rivaroxaban—6.5%, edoxaban—3.5%, dabigatran—2.3%, and apixaban—1.3%) [44]. Rivaroxaban was approved by the FDA in 2011 [45] and had the highest ADR reporting rate among DOACs. Even if no correlation can be made between the number of EV reports and drug sales, it still seems that the upward trend of rivaroxaban sales, and its first position worldwide with sales of 7.6 billion USD from a total of 17.2 billion USD recorded for DOACs [46], could justify the highest number of reports uploaded in EV. The regional sales distribution of this drug shows that more than EUR 2.25 billion was generated in Europe at that time [47]. The same trend was registered in a sales forecast for Europe, published in 2016, which showed that rivaroxaban would have sales of EUR 2.6 billion by 2022, maintaining its position in the lead of the European DOAC market until then [48].

Regarding the volume of the market, worldwide, approximately 74 million patients had rivaroxaban prescribed, thus becoming the most prescribed DOAC in 2020 [47]. Italy and Denmark showed an increase in DOAC use between 2010 and 2018 [49]. In 2018 in the Netherlands, apixaban and rivaroxaban were the most used DOACs [50]. Studies related to atrial fibrillation show that apixaban followed by rivaroxaban were the most prescribed DOACs from 2017 in countries such as Belgium, France, Germany and UK [51].

Thus, comparing the increasing trend of the DOACs market with the reported ADRs, it can be considered that the quantity of prescriptions has augmented [52]. Moreover, according to an EMA report published in 2020, rivaroxaban was the most used DOAC in the majority of databases consulted for this assessment report [53]. From the total number of DOAC dosing errors (*n* = 6825), 51.56% represented underdose errors (*n* = 3519), 18.54% overdose errors (*n* = 1265), and 29.90% improper dose errors (*n* = 2041) (Figure 1a). According to different studies, underdosing is one of the most prevalent ME of DOACs [28,54]. Moreover, a study performed on a large database including patients with AF showed a strong tendency to administrate DOAC underdoses (13.3%) in the absence of an indication to reduce the dose. On the other hand, the same study noted a high proportion of overdoses used by patients with renal indication for decreasing the doses [36]. Our results show that dosing errors reported in the EV database were most common for rivaroxaban (*n* = 3687, 54.02%), followed by apixaban (*n* = 2294, 33.61%). These errors were similarly reported for dabigatran (*n* = 427, 6.26%) and edoxaban (*n* = 417, 6.11%) (Figure 1b).

Underdosing (*n* = 3519) was the most common ADR associated with rivaroxaban (*n* = 1703, 48.39%), apixaban (*n* = 1637, 46.52%), dabigatran (*n* = 43, 1.22%), and edoxaban (*n* = 136, 3.87%). A variation between 0.07% (dabigatran) and 2.32% (apixaban) (Figure 2) was observed regarding the distribution of this ADR in the total number of reports. Several research groups have also underlined the frequent off-label underdosing for DOACs [31,55,56,57].

In addition, 2041 ADRs were related to improper doses: 1569 for rivaroxaban (76.88%), 225 for edoxaban (11.02%), 175 for apixaban (8.57%), and 72 for dabigatran (3.53%). Out of the total ADRs reported, improper dosing was most frequently observed for edoxaban (2.41%), and less frequently for dabigatran (0.12%) (Figure 2).

Overdosing was the least common dosing error reported in EV (*n* = 1265): apixaban (*n* = 482, 38.10%), rivaroxaban (*n* = 415, 32.81%), dabigatran (*n* = 312, 24.66%), and edoxaban (*n* = 56, 4.43%). Out of the total ICSRs, the overdose reports had a frequency between 0.32% (dabigatran) and 0.68% (apixaban) (Figure 2).

Figure 3 showed the highest ratio of fatal outcome related to dabigatran (9.94%—overdosing errors) and edoxaban (8.93%—overdosing errors). Dabigatran and edoxaban had the highest proportion of NR/NS outcomes from the total ADRs (Figure 4). Thus, apixaban and rivaroxaban had a lower ratio of fatal or NR/NS outcomes.

Overdosing of DOACs or antiplatelet drugs is responsible for a variety of bleeding disorders [32,58,59]. Figure 5 represents the probability of reporting DOAC overdosing compared to antiplatelet drugs. Thus, the results of the disproportionality analysis do not show statistical differences between DOAC reports and other AC drugs or most antiplatelet drugs, except for ticagrelor and ticlopidine. Underdosing of AC or antiplatelets has led to an increased rate of cardiovascular hospitalization and an increased risk of all-cause mortality, stroke or myocardial infarction [60,61]. Compared to the majority of AC or antiplatelets drugs (except for certoparin, bivalirudin, ticlopidine, and prasugrel), the reporting of ADRs related to the underdosing of rivaroxaban, apixaban and edoxaban had a higher rate. No difference was registered between the probability of reporting underdoses for dabigatran compared to all ACs or antiplatelet drugs (Figure 6). In the present study, for improper dosing errors of dabigatran and apixaban, no difference in reporting was noted compared to other ACs or antiplatelets. A significant difference between rivaroxaban and edoxaban versus reports for most AC or antiplatelet drugs was observed (Figure 7).

Patients’ satisfaction determines the adherence to their treatment; thus, the therapy outcome is improved [62]. Thus, many of the mentioned medication errors could be prevented if health professionals would become more involved in patient education and satisfaction [63,64]. The collaboration and the complementarity of knowledge of physicians and pharmacists may offer a reliable solution to DOAC dose management [63]. The counselling offered by pharmacists empowers the patient and helps avoid or promptly identify adverse effects [64].

### Limitations of the Study

This study comprises the PTs related to dosing errors available in EV. Since not all the existing ones and ADR reports related to DOACs are available through this database, the precision of the study might be limited. The underreporting of ADRs is a widespread phenomenon, the number of reported ADRs being below the number of ADRs that occur. Statistics available through the EV database, or other pharmacovigilance systems, only show data on ADRs reported.

The EV database does not comprise information regarding the use of DOACs. Moreover, quantitative data based on DOAC usage in the EEA countries that participated in the EV database have restricted access. The scientific literature available provides very limited information regarding DOAC sales or number of prescriptions, and the ones that are available are lacking in homogeneity (e.g., different therapeutic indications, patients’ category, country/region, reporting period). Correlations based on this incomplete and variable information would only be speculative. The reported ADRs or case reports depend on several factors, such as awareness of the patient, the nature of the reaction, the administration of other drugs, the conditions in which the drug was used, etc. The safety profiles observed could be limited in precision; thus, the data cannot be used to determine the frequency of ADRs because the spontaneous cases reported in EV are based on a suspicion of causality, which does not necessarily mean that a causal relationship has been established.

The results of the current descriptive analysis do not allow for the identification of the causes regarding the highest frequency of drug error reports on rivaroxaban, one of which might be represented by the possible highest usage of this drug compared to other DOACs.

The calculated ROR cannot be used for the quantitative determination of ADR risk for DOACs because it only indicates the potential safety issues that might occur.

## 4. Materials and Methods

### 4.1. Study Design

A descriptive analysis was performed based on the ICSR from the EV database. The analyzed ICSR included suspected ADRs related to DOAC dosing errors (overdose, underdose, improper dose). Reports uploaded until 3 December 2022 (rivaroxaban from 20 February 2008, apixaban from 6 February 2009, dabigatran from 25 April 2005, and edoxaban from 17 February 2014) were extracted from the electronic database available at https://www.adrreports.eu/, accessed on 5 December 2022. The total number of ADRs for each PT was determined. Subsequently, the number of “fatal” ADRs and “NR/NS” ADRs was noted for each evaluated PT since the date of the first report registered in EV. Causality and severity were not limiting criteria for the inclusion of ICSR in EV [65]. The Pearson correlation coefficient was calculated to evaluate the correlation between each category of ADRs (overdosing errors, underdosing errors, modified dose errors, fatal dosing errors, and NR/NS dosing errors) and total ADRs reported in EV.

### 4.2. Material

According to the Medical Dictionary for Regulatory Activities (MedDRA) terminology, MEs related to drug dosing are grouped under “injury, poisoning and procedural complications” SOC (system organ classes). Out of a total number of 91 PTs selected from MedDRA, the ADRs reported in EV for DOACs were related to only 24 PTs. These PTs were classified into three categories: (a) improper dose; (b) overdose; (c) underdose (Table 3). PTs referring to dosing error but not associated with overdosing or underdosing were included in the improper dose category (a). PTs containing “dose omission” were considered underdosing errors. PTs referring to intentional overdose, underdose or improper dose were not included (according to the EV recommendations). At the date of the study, EV registered ICSR for dabigatran, rivaroxaban, apixaban and edoxaban.

### 4.3. Disproportionality Analysis

Subsequent to the descriptive analysis, a disproportionality analysis was performed. The ROR was calculated, and a 95% CI was considered. According to the recommendations, data reported in the EV system could be analyzed if the individual cases were classified into four categories and two dichotomous variables (a two-by-two contingency table) [66]. The ROR was established for the three types of drug dose errors (categories a, b, c, Table 3). The total number of cases for each category resulted by summing the number of cases reported for each PT included in the corresponding category. In order to calculate the ROR, it is recommended to use comparators from common therapeutic areas [67]. Thus, antiplatelets and other AC drugs (Table 4) were used as drug references for ADRs reported for dabigatran, rivaroxaban, apixaban, and edoxaban. All reports were considered regardless of drug role (suspect, interacting, or concomitant). According to EMA guidelines, disproportionality signals were defined when cases were ≥5 and ROR was statistically >1 (lower limit of 95% CI > 1) [66]. The analysis was performed with MedCalc Software Ltd. Odds ratio calculator [65]; https://www.medcalc.org/calc/odds_ratio.php (accessed on 5 December 2022) (Version 20.123). Data extraction for the disproportionality analysis was performed on 3 December 2022.

### 4.4. Ethics

Data were reported anonymously by healthcare professionals or non-healthcare professionals, including patients and marketing authorization holders, and patients could not be identified on the portal. Thus, no ethical approval was necessary.

## 5. Conclusions

DOACs have been on the market since 2008 and have offered a safer alternative to vitamin K antagonists and heparins. This retrospective analysis of ADRs related to DOAC dosing errors showed that underdosing is still the most frequently encountered dosing error (51.56%), as opposed to overdose errors (18.54%). Only four (rivaroxaban, apixaban, edoxaban, and dabigatran) of the five current representatives of the DOAC class were mentioned in ADR reports from EV. Out of the total ADRs reported for ACs, 30.7% were related to rivaroxaban. An interdisciplinary medical team including a clinical pharmacist can offer a complete solution to identify and address dose errors at any level, including drug prescribing, dispensing and administration.

## Figures and Tables

**Table 1:** Total ADRs reported in EV related to DOACs.

Drug	Total ADRs	% of Total
Dabigatran	57,627	13.49%
Rivaroxaban	131,182	30.70%
Apixaban	70,424	16.48%
Edoxaban	9318	2.18%
Other ACs	158,767	37.15%
	427,318	

**Table 2:** The correlation coefficient between ADR categories and total ADRs reported in EV.

Improper Dose Errors—Total ADRs	Overdosing Errors—Total ADRs	Underdosing Errors—Total ADRs	Fatal Dosing Errors— Total ADRs	NR/NS Dosing Errors—Total ADRs
0.8193	0.7801	0.7723	0.7652	−0.6455
0.1807	0.2199	0.2277	0.2348	0.355

**Table 3:** PTs for MEs related to drug doses.

Category	ADR	PT
a	Improper dose	Dose calculation error
		Dose calculation error associated with device
		Drug dose titration not performed
		Drug titration error
		Incorrect dosage administered
		Incorrect dose administered
		Incorrect dose administered by device
		Incorrect dose administered by product
		Incorrect product dosage form administered
		Product dosage form confusion
		Wrong dosage formulation
		Wrong dose
b	Overdose	Accidental overdose
		Extra dose administered
		Overdose
		Prescribed overdose
c	Underdose	Accidental underdose
		Drug dose omission by device
		Incomplete dose administered
		Prescribed underdose
		Product dose omission
		Product dose omission in error
		Product dose omission issue
		Underdose

**Table 4:** Drugs used as comparators for disproportionality analysis.

Drug Category	Drug	Pharmacologic Class
Anticoagulant drugs	Heparin	Unfractionated heparin
	Dalteparin	Low molecular weight heparin
	Enoxaparin	Low molecular weight heparin
	Nadroprin	Low molecular weight heparin
	Tinzaparin	Low molecular weight heparin
	Reviparin	Low molecular weight heparin
	Parnaparin	Low molecular weight heparin
	Certoparin	Low molecular weight heparin
	Bemiparin	Low molecular weight heparin
	Semuloparin	Low molecular weight heparin
	Deligoparin	Low molecular weight heparin
	Fondaparinux	Parenteral direct factor Xa inhibitor
	Danaparoid	Heparinoid
	Pentosan polysulphate	Heparinoid
	Argatroban	Parenteral direct thrombin inhibitor
	Desirudin	Parenteral direct thrombin inhibitor
	Lepirudin	Parenteral direct thrombin inhibitor
	Bivalirudin	Parenteral direct thrombin inhibitor
	Warfarin	VKA
	Acenocoumarol	VKA
	Dicumarol	VKA
	Phenindione	VKA
	Anisindione	VKA
	Fluindione	VKA
Antiplatelet drugs	Clopidogrel	Platelet aggregation inhibitors
	Ticagrelor	Platelet aggregation inhibitors
	Prasugrel	Platelet aggregation inhibitors
	Ticlopidine	Platelet aggregation inhibitors
	Cilostazol	Phosphodiesterase type 3 inhibitor
	Triflusal	Platelet aggregation inhibitors
	Abciximab	Glycoprotein platelet inhibitors
	Eptifibatide	Glycoprotein platelet inhibitors
	Tirofiban	Glycoprotein platelet inhibitors
	Vorapaxar	Protease-activated receptor-1 antagonists
	Dipyridamole	Nucleoside transport and phosphodiesterase type 3 inhibitor
	Dipyridamole and Acetylsalicylic acid	Combination
	Acetylsalicylic acid and Clopidogrel	Combination

## Data Availability

Data is contained within the article.

## Abbreviations

ABC	abciximab
AC	anticoagulant drugs
ACE	acenocoumarol
ACL	combination of acetylsalicylic acid and clopidogrel
ADRs	adverse reactions
AF	atrial fibrillation
API	apixaban
ARG	argatroban
BEM	bemiparin
BIV	bivalirudin
CER	certoparin
CI	confidence interval
CIL	cilostazol
CLO	clopidogrel
DAB	dabigatran
DAL	dalteparin
DAN	danaparoid
DAS	combination of dipyridamole and acetylsalicylic acid
DIP	dipyridamole
DOACs	direct oral anticoagulant drugs
EDO	edoxaban
EEA	European Economic Area
EMA	European Medicines Agency
ENO	enoxaparin
EPT	eptifibatide
EV	EudraVigilance
FLU	fluindione
FON	fondaparinux
HEP	unfractionated heparin
ICSR	Individual Case Safety Report
LEP	lepirudin
LMWHs	low molecular weight heparins
ME	medication errors
MedDRA	Medical Dictionary for Regulatory Activities
NAD	nadroparin
NR/NS	not recovered/not resolved
PRA	prasugrel
PT	preferred term
RIV	rivaroxaban
ROR	reporting odds ratio
SOC	system organ classes
TCL	ticagrelor
TIC	ticlopidine
TIN	tinzaparin
TIR	tirofiban
VKAs	vitamin K antagonists
WAR	warfarin
